# Life Cycle Assessment of the Sustainability of Enhancing the Photodegradation Activity of TiO_2_ with Metal-Doping

**DOI:** 10.3390/ma13071487

**Published:** 2020-03-25

**Authors:** Sónia Fernandes, Joaquim C.G. Esteves da Silva, Luís Pinto da Silva

**Affiliations:** 1 Chemistry Research Unit (CIQUP), Faculty of Sciences of University of Porto, R. Campo Alegre 697, 4169-007 Porto, Portugal; soniaf91991@gmail.com (S.F.);; 2 LACOMEPHI, GreenUPorto, Department of Geosciences, Environmental and Territorial Planning, Faculty of Sciences of University of Porto, R. Campo Alegre 697, 4169-007 Porto, Portugal

**Keywords:** life cycle assessment, TiO_2_ photocatalysts, photodegradation, metal-doping, engineered nanomaterials

## Abstract

While TiO_2_ nanoparticles have shown potential as photocatalysts in the degradation of organic contaminants, their inability to absorb efficiently visible light has limited their industrial application. One strategy for solving this problem is monodoping TiO_2_ photocatalysts with transition metals, which has worked in the degradation of several pollutants. However, it is not clear if this improvement is enough to offset the potential environmental impacts of adding metal ions to the synthesis of TiO_2_. Herein, we have used Life Cycle Assessment (LCA) to determine the sustainability of monodoping TiO_2_ with transition metals (Fe, Co, Mn and Ni, with a 1% weight ratio) to enhance the photocatalytic properties of the photocatalyst toward the degradation of Carbamazepine and Methyl Orange, under UV-A and visible light irradiation. We found that the addition of transition-metals has no significant effect on the environmental impacts associated with the synthesis of TiO_2_, when a weight-based functional unit was considered. However, when photocatalytic activity was considered, major differences were found. Thus, our results demonstrate that the sustainability of monodoping with different transition metals is solely determined by their ability to enhance (or not) the photocatalytic activity of TiO_2_. Our data also demonstrated that isopropyl alcohol constitutes a critical point in the synthesis of TiO_2_ photocatalysts, with ethanol being a potential substitute.

## 1. Introduction

Water pollution is an important environmental issue, as pollutants can come from different sources, e.g., pharmaceutical, pesticides, herbicides, textiles dyes, resins, and phenolic compounds, among others [1,2,3]. This has led to concerns regarding the depletion of underground water resources and suitable wastewater management [1].

Advanced Oxidation Processes (AOPs) constitute an innovative water treatment technology that consists of the production of highly reactive transitory species for the degradation of organic pollutants [4,5]. Heterogeneous photocatalysis employing inorganic oxides such as TiO_2_, ZnO, and Fe_2_O_3_ has been shown to be able to efficiently degrade a broad range of pollutants without generating harmful intermediates [6]. Among them, titanium dioxide (TiO_2_) has received the most attention, mainly in the Research and Development (R&D) of photocatalysis technology [6]. 

TiO_2_ has been commercially produced as a white pigment since the early 20th century. There is now also a market for anatase-phase TiO_2_, i.e., particles that fall into the nano range, with diameters between 1 and 100 nm [7]. The main advantages of TiO_2_ photocatalyst are its low cost, chemical and thermal stability, strong oxidizing power, high photocatalytic activity, and its environmental friendliness [8]. However, the large bandgap (~3.20 eV) of TiO_2_ means that it is only able to absorb in ultraviolet (UV) wavelengths, greatly inhibiting its industrial applications under visible-light irradiation [4,9,10]. Given this, several authors have tried to develop visible-responsive TiO_2_ photocatalysts by doping them with either metal ions and/or nonmetal ions, or by the creation of hetero-junctions with other semiconductors [4,11,12]. Among these strategies, transition-metal doping has been shown to be quite effective [12,13,14].

Our group has been active in this field and has developed several TiO_2_ nanomaterials monodoped with different metal ions, e.g., iron (Fe), cobalt (Co), manganese (Mn) and nickel (Ni) [15,16]. The photocatalytic properties of these nanoparticles were evaluated for the photo-degradation of Methyl Orange (MO) [15] and Carbamazepine (CBZ) [16] and compared with the activity shown by undoped TiO_2_. More specifically, undoped TiO_2_ and monodoped Co–TiO_2_, Mn–TiO_2_, and Ni–TiO_2_ were employed in the photo-degradation of MO under UV-A and visible light irradiation [15]. We found that while monodoping decreases the UV-A-triggered photo-degradation (from 95.6% MO conversion for undoped TiO_2_ to 14.6–49.3% for doped nanomaterials), monodoping with Co significantly increased the photo-degradation capabilities under visible light (16.3% for undoped TiO_2_ but 33.3% for Co–TiO_2_) [15]. Unfortunately, both monodoping with Mn (conversion of 6.8%) and Ni (conversion 13.8%) negatively affected the photocatalytic properties of TiO_2_ in the degradation of MO [15]. 

We also tested undoped TiO_2_ and monodoped Fe–TiO_2_ and Co–TiO_2_ in the UV-A and visible light-based photodegradation of CBZ [16], a persistent organic pollutant that poses a threat to water quality [14,17]. Quite encouragingly, Fe–TiO_2_ presented better results for the removal of CBZ than undoped TiO_2_ under both UV-A (96.9% versus 70.1%) and visible light (12.5% versus 7.9%) irradiation [16]. Moreover, while Co–TiO_2_ presented worse results than undoped TiO_2_ under UV-A irradiation (34.2% versus 70.1%), it also presented better results under visible light irradiation (10.8% versus 7.9%) [16].

Thus, our results also support the notion that doping TiO_2_ with transition-metal ions is an efficient strategy to improve the visible light-based photo-degradation activity of this nanomaterial. However, it should be noted that while this appears to be a strategy with significant potential going forward, it is not clear how metal ion doping affects the sustainability of the fabrication and usage of the TiO_2_ photocatalysts, and if the photocatalytic benefits derived from this approach can offset the potential environmental impacts. This is a glaring lack of knowledge, because if the negative environmental impacts introduced by including transition metal ions in the synthesis of TiO_2_ photocatalysts offset the improvement of photocatalytic properties under visible light irradiation, the use of this particular doping strategy must be discouraged. 

The importance of elucidating this topic is also supported by the fact that the production of nanoparticles has been shown to be more energy- and material-consuming than that of fine chemicals and pharmaceuticals [18,19]. Moreover, other studies demonstrated that the energy and chemicals used during the synthesis of nanoparticles contribute significantly to their environmental impact [20,21,22,23]. Therefore, evaluating the sustainability of the transition metal doping strategy in the synthesis and photocatalytic usage of TiO_2_ should be a priority.

Herein, the aim of this work is to determine the sustainability of monodoping TiO_2_ with different transition metal ions (Fe, Mn, Co, and Ni) while considering performance in the UV-A and visible light-based photo-degradation of MO and CBZ [15,16]. The sustainability and associated environmental impacts of each process were analyzed with a life cycle assessment (LCA) study of each synthesis. LCA is the most suitable approach to address this issue, as it makes it possible to quantify the environmental impacts of a given system during its life cycle [24,25]. Also, LCA has already been used with success in the environmental evaluations of different nanomaterials, such as undoped TiO_2_ [26], silver nanoparticles [20], copper nanoparticles [21], carbon nanotubes [22], magnetic nanoparticles [23], and carbon dots [27,28].

## 2. Materials and Methods 

This section is divided into five subsections as described below.

### 2.1. Scope and System Boundaries

This cradle-to-grave study aims to quantify and compare the environmental impacts of undoped and transition-metal-monodoped TiO_2_ photocatalysts in order to achieve improved pollutant degradation with reduced environmental impact. This work aims to study the laboratory-scale manufacturing stage of target nanoparticles, and considers the direct emissions from TiO_2_ production and the indirect impacts associated with upstream resource extraction and energy generation.

**Scheme 1:**
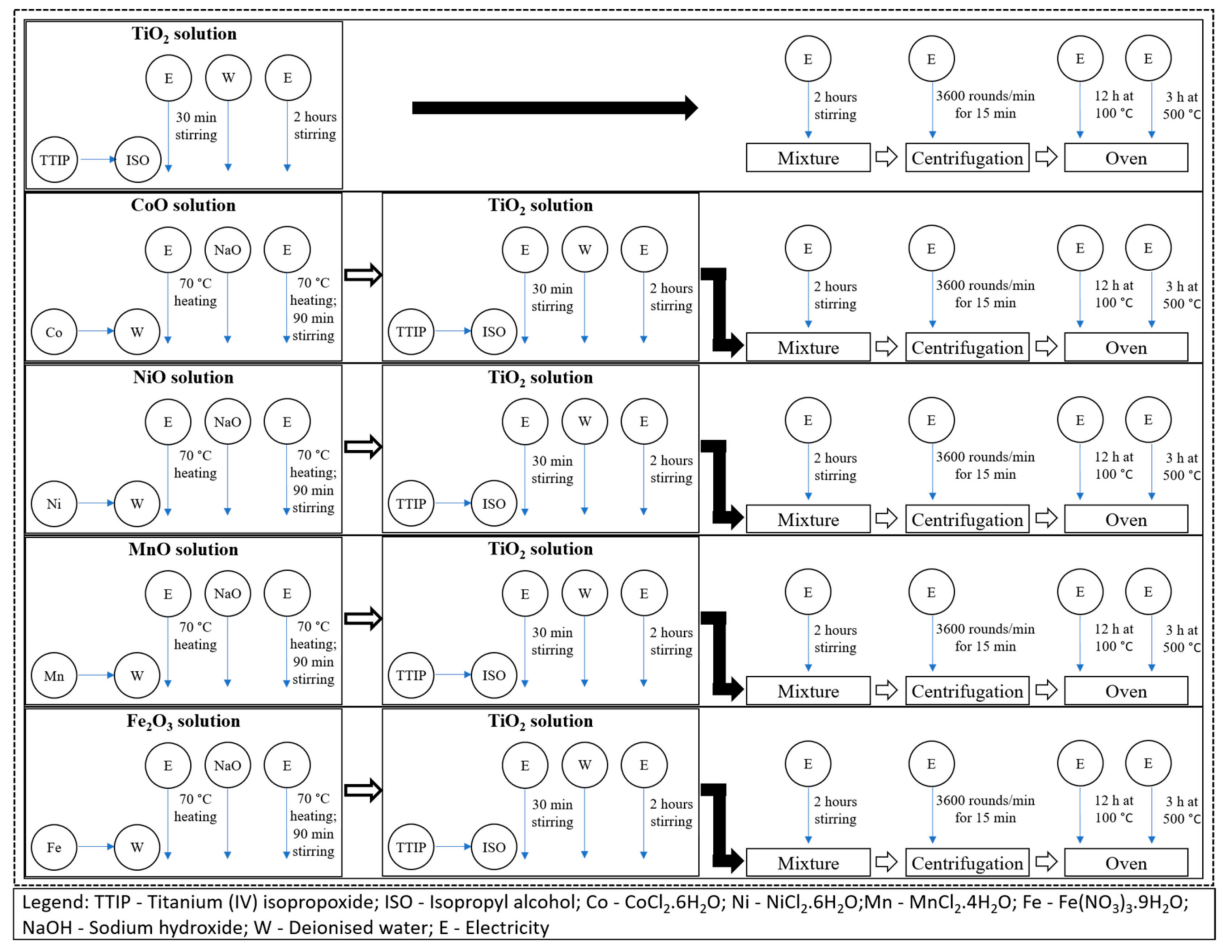
Diagram for undoped and doped TiO_2_ photocatalysts synthesis.

This study uses the fabrication routes employed previously by us for the synthesis of undoped TiO_2_ and monodoped Fe–TiO_2_, Co–TiO_2_, Mn–TiO_2_, and Ni–TiO_2_ [13,14], which are described in detail below (Scheme 1). The environmental impacts were compared initially using a weight-based functional unit of 1 kg of nanoparticles, given that this can be used to compare the production of an equivalent amount of nanomaterial. These impacts were subsequently normalized by the photo-degradation efficiency (in %) of the photocatalysts toward the removal of either MO and CBZ under UV-A and visible light-based irradiation, as determined previously by us [15,16]. Such an approach is needed because a weight-based function might not account for the possibility that a more resource-intensive synthesis may be justified later in the use stage (given the improved functionality).

### 2.2. Synthesis Routes 

The materials used in the procedure for the synthesis of all TiO_2_ catalysts under study are detailed in the Supplementary Materials Table S1. This study considers the synthesis routes employed previously by us for the preparation of five TiO_2_ photocatalysts, i.e., one undoped and four monodoped with transition metals (Co, Ni, Mn or Fe) [15,16]. Pure TiO_2_ was synthesized using the sol-gel method, and metal-doped TiO_2_ with the sol-gel and precipitation methods combined. The amounts considered in this LCA are related to 1 kg of catalyst, and metal-doped photocatalysts constitute 1 weight percent (wt.%) of the doping elements (Co, Ni, Mn or Fe) [15,16].

Briefly, the undoped TiO_2_ catalyst and TiO_2_ in the doped catalysts were prepared by dissolving titanium (IV) tetraisopropoxide (TTIP) in isopropyl alcohol (ISO) under continuous stirring at room temperature for 30 min. Subsequently, water was added dropwise, and then the mixture was stirred continuously for 2 h at room temperature. The obtained dispersion was centrifuged at 3600 rpm/min for 15 min, washed with deionized water, filtered, and dried for 12 h at 100 °C in an oven. Finally, the obtained material was ground and calcined for 3 h at 500 °C [15].

For the studied metals, the dopant precursor (CoCl_2_, MnCl_2_, NiCl_2_, and Fe(NO_3_)_3_) was dissolved in deionized water while stirring; then, an aqueous solution of NaOH at 70 °C was added dropwise. The solutions were continuously stirred for 90 min and then the obtained solutions were added dropwise to the colloidal solutions of the TiO_2_, which was prepared as mentioned above. The resulting mixture was continuously stirred for 2 h and centrifuged for at 3600 rpm for 15 min. Then it was filtered, washed with deionized water, and dried in an oven for 12 h at 100 °C. The obtained nanoparticles were ground and calcined at 500 °C for 3 h [15,16].

### 2.3. Life Cycle Inventory Data

The environmental impact relative to the syntheses of TiO_2_ photocatalysts was evaluated based on inventory data from laboratory-scale synthesis procedures found in the Ecoinvent® (Zurich, Switzerland) 3.5 database. The foreground system of the synthesis procedure consists of chemicals used as raw materials and electricity used in the fabrication process (heating plate, centrifuge, oven). The processes and chemicals included here were modeled with the following data present in the Ecoinvent® 3.5 database (GLO: global; RER: regional market for Europe; and PT: Portugal):

- Isopropanol {GLO}
- Sodium hydroxide, without water, in 50% solution state {GLO};
- Cobalt {GLO};
- Nickel sulfate {GLO};
- Manganese sulfate {GLO};
- Iron (III) chloride, without water, in 40% solution state {GLO};
- Water, deionized, from tap water;
- Electricity, medium voltage {PT}.

TTIP was not included in this database, so it was introduced based on [29], as detailed in the Supporting Information on Table S2. It should be noted that while the dopant metal reagents used experimentally in the synthesis of monodoped TiO_2_ were in the form of metal chlorides [15,16], due to the absence of these chemicals in this database, we were limited to other forms of these metal dopants (mainly metal sulfates). Our rationale for this choice was that the identity and oxidation state of the metal were expected to be more relevant for the synthesis of metal-doped TiO_2_, and not the identity of the anionic nonmetal compounds of the metal precursor.

The amount of chemicals and electricity used are described in Section 2.2 and in the Supplementary Materials. The dataset used for electricity describes the available electricity data on the medium voltage level in Portugal for the year 2014, as described in the Ecoinvent® 3.5 database. The electricity consumption considered here is what is required to use the heating plate, oven, and centrifuge. The heating and stirring were done using a Normax Nx1200 Analogical magnetic stirrer (Marinha Grande, Portugal) with heating, which has a power consumption of 500 W. Centrifugation was done with a Hettich MIKRO 220R (Tuttlingen, Germany) with 3.8 A and a maximum power consumption of 874 W with a maximum power factor (1). The oven used was a Furnace 47900 from Thermolyne, which has a power consumption of 1000 W.

### 2.4. Environmental Impact Assessment

The present LCA study is based on a cradle-to-grave approach, i.e., from the production of precursor materials to their disposal. Environmental impact was modeled using the ReCiPe 2016 V1.03 endpoint method, Hierarchist version, [30], which evaluates three categories of potential impacts (Human Health, Ecosystems, and Resources). In the Human Health subsection, Global Warming–Human Health (GW–HH), Stratospheric ozone depletion (SO), Ionization Radiation (IR), Ozone formation–Human Health (OF–HH), Fine Particulate Matter formation (FPM), Human Carcinogenic toxicity (HC), Human Non-Carcinogenic toxicity (HNC) and Water Consumption–Human Health (WC–HH). Ecosystems potential impacts evaluated Global Warming–Terrestrial Ecosystems (GW–TE), Global Warming–Freshwater Ecosystems (GW–FE), Ozone Formation–Terrestrial Ecosystems (OF–TE), Terrestrial acidification (TA), Freshwater Eutrophication (FE), Marine Eutrophication (ME), Terrestrial EcoToxicity (TET), Freshwater EcoToxicity (FET), Marine EcoToxicity (MET), Land Use (LU), Water Consumption–Terrestrial Ecosystem (WC–TE) and Water Consumption–Aquatic Ecosystems (WC–AE) were assessed. In the Resources subsection, Mineral Resource scarcity (MR) and Fossil Resource scarcity (FR) were assessed. The LCA study was performed using SimaPro 9.0.0.48 software (Amersfoort, The Netherlands).

### 2.5. Sensitivity Analysis

Given that doped TiO_2_ photocatalysts are in the early stages of development on a laboratory scale, the environmental impact of their fabrication is still uncertain. To this end, a sensitivity analysis was performed in order to reduce such uncertainties for scaled-up production [31]. This was done by considering “what-if” scenarios [32], that is, by changing the amount and type of materials and electricity employed in the synthesis of these photocatalysts.

## 3. Results

### 3.1. Synthesis Comparison by Weight

**Figure 1:**
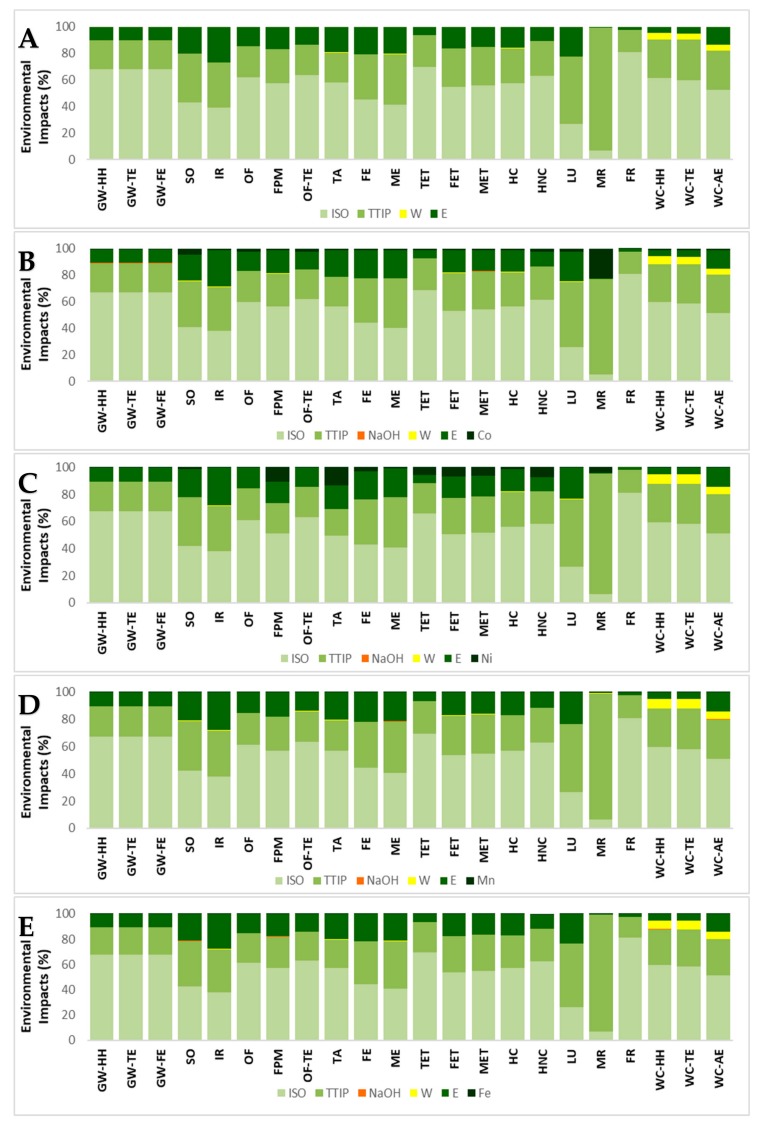
Relative environmental impacts of synthesis of the undoped TiO_2_ photocatalyst (**A**), Co-doped TiO_2_ photocatalyst (**B**), Ni-doped TiO_2_ photocatalyst (**C**), Mn-doped TiO_2_ photocatalyst (**D**), and Fe-doped TiO_2_ photocatalyst (**E**).

The potential environmental impacts of the undoped and metal-doped TiO_2_ catalysts under study are shown in Figure 1. From this analysis, it was possible to see that the major contributor in almost all the environmental impact categories is ISO. The categories of Land Use (LU) and Mineral Resource Scarcity (MR) are the exceptions, in which TTIP is the highest contributor. Deionized water appears to be a comparatively negligible contributor, even in impact categories related to water consumption. These results are in line with the literature; a recent work by Wu et al. [33] demonstrated that different precursors have the greatest environmental impact for chemical synthesis routes, such as the sol-gel approach studied here [15,16].

Interestingly, no significant differences appear to exist between the syntheses of undoped and monodoped TiO_2_ photocatalysts (Figure 1). In fact, only the addition of Co and Ni precursors leads to some differences regarding the resulting environmental impact, namely, the Co precursor is the second major contributor concerning Mineral Resource Scarcity (MR), while the addition of nickel precursors implies a slight contribution from this material for Fine Particulate Matter formation (FPM) and Terrestrial Acidification (TA). Thus, our results indicate that the addition of doping metals does not significantly affect the sustainability of TiO_2_ fabrication.

**Figure 2:**
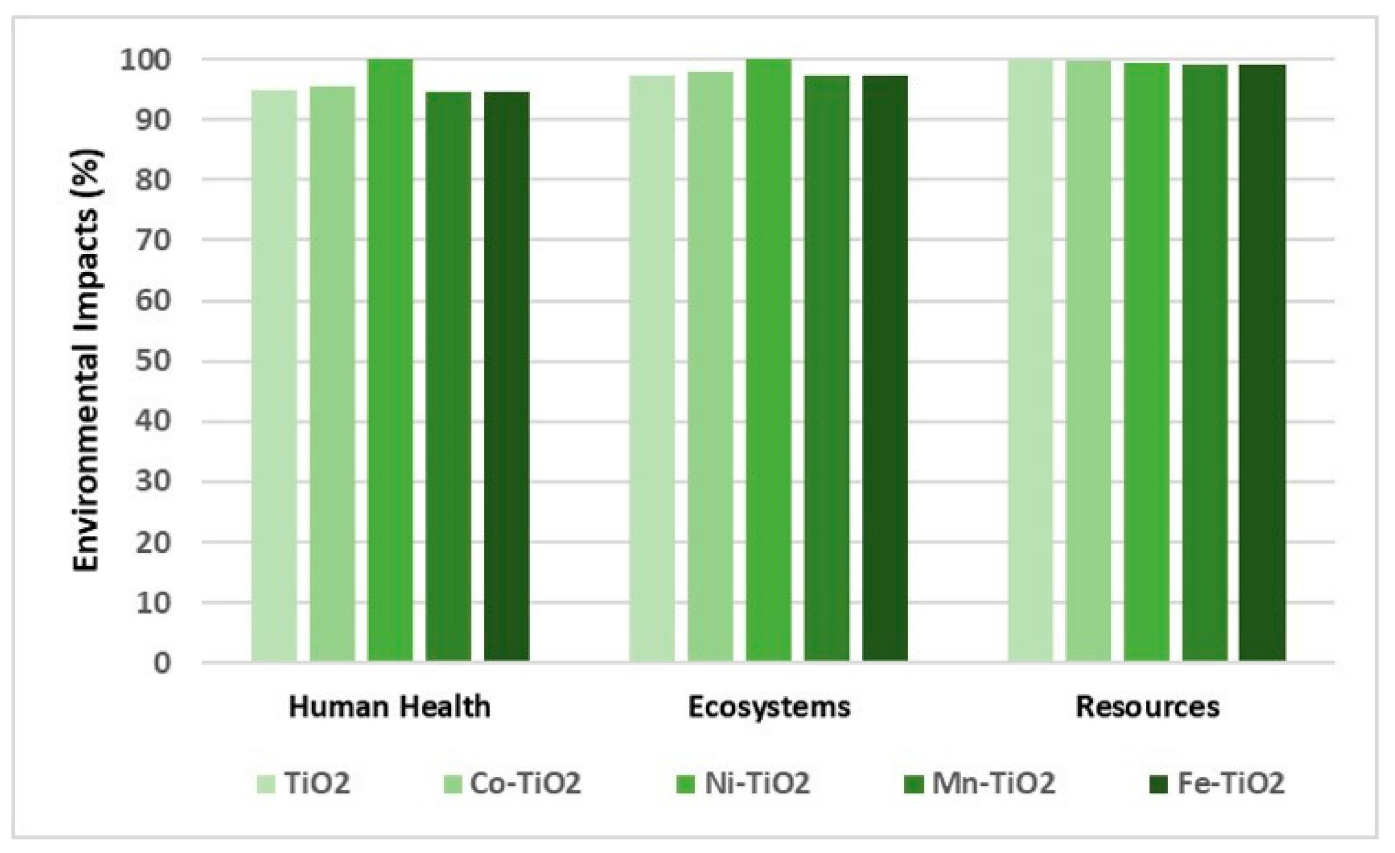
Relative environmental impact of different TiO_2_ photocatalysts, considering a weight-functional unit of 1 kg.

This was confirmed by comparing the environmental impacts associated with the five nanomaterials, as presented in Figure 2. The differences are negligible in the Resources category, and quite low in the Human Health and Ecosystem categories. In fact, for the latter two categories, only Ni–TiO_2_ induces slightly higher impacts, while the other four nanoparticles present identical impacts. Nevertheless, the small differences between Ni–TiO_2_ and the other nanoparticles are probably not significant. 

### 3.2. Synthesis Comparison by Photocatalytic Activity

**Figure 3:**
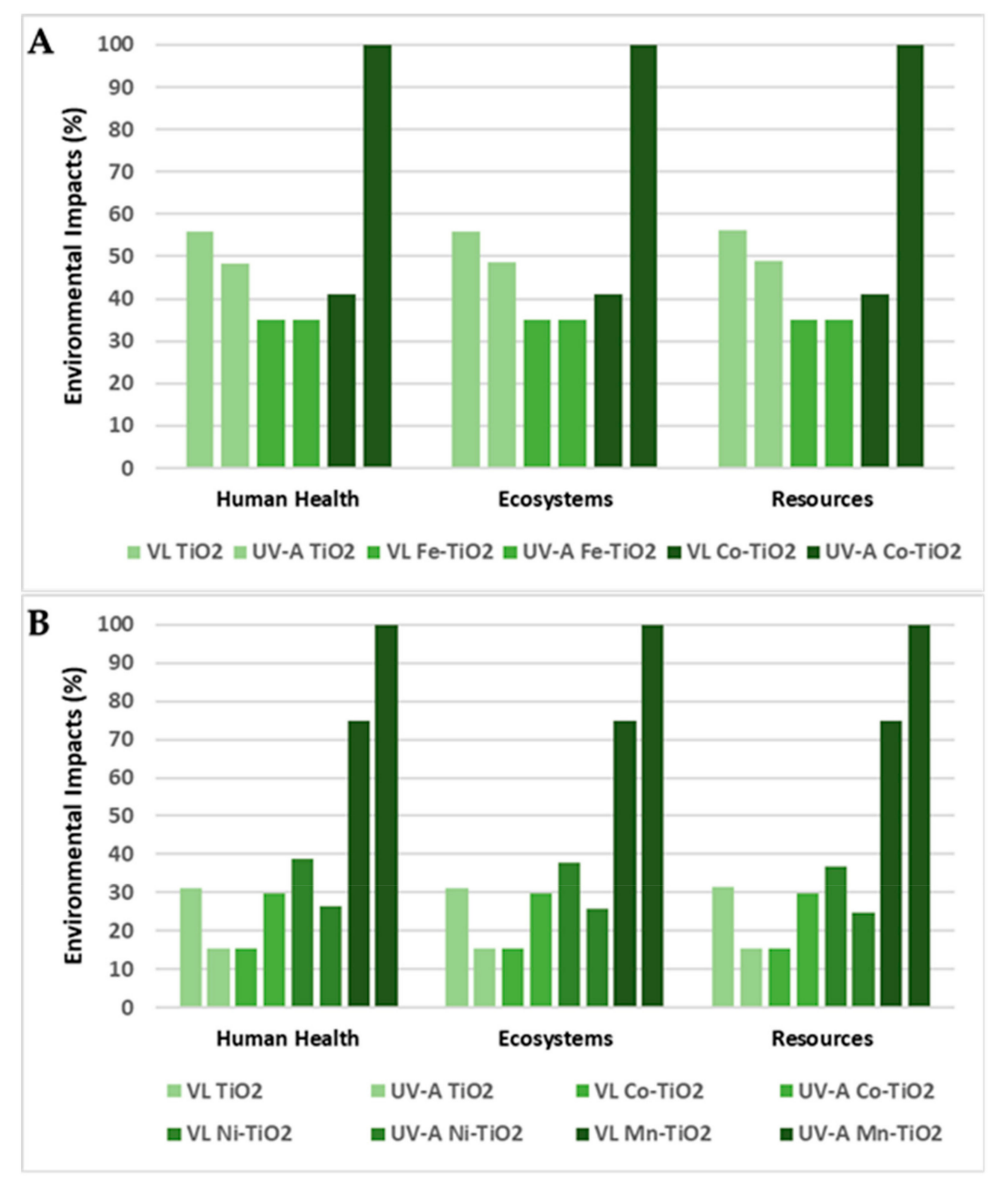
Relative environmental impacts of different TiO_2_ photocatalysts synthesis for CBZ (**A**) and MO (**B**), considering photocatalytic activity as a functional unit, under either UV-A or visible-light (VL) irradiation.

The environmental profiles (Figure 3) were also evaluated for the synthetic routes of the different TiO_2_ photocatalysts according to their photocatalytic activity in the degradation of either CBZ or MO, both under UV-A and visible light irradiation (Table 1). 

The rescaling was done by considering the photo-degradation of each pollutant under each type of light source, i.e., the function-based functional unit is presented as a ratio, $\frac{Photocatalytic\;activity_{NP}^{REF}}{Photocatalytic\;activity_{NP}^{}}$, in which the photocatalytic activity of each nanoparticle (NP) consists of the removal efficiency (in %) of the target pollutant as determined previously [15,16], while $Photocatalytic\;activity_{NP}^{REF}$ refers to the nanoparticles with higher photocatalytic activity for each case (thus acting as a reference). Therefore, higher functional units indicate lower photocatalytic activity and higher associated environmental impact.

For CBZ (Figure 3A), significant differences exist according to the function-based functional unit, which illustrates the importance of considering the photocatalytic activity of CBZ degradation. More specifically, there is little difference for the weight-based functional unit (Figure 2). However, for the functional unit based on photocatalytic activity, Co–TiO_2_ clearly leads to greater potential impacts than both Fe–TiO_2_ and undoped TiO_2_, under both UV-A and visible light irradiation. Interestingly, Fe–TiO_2_ has a lower environmental impact than undoped TiO_2_ under both types of light sources, which can be explained by the higher photocatalytic activity of the former nanomaterial. Thus, our analysis demonstrates that Fe monodoping is a sustainable strategy for improving the photocatalytic activity of TiO_2_ in the degradation of CBZ.

For MO (Figure 3B), Co–TiO_2_ is the nanoparticle with the lowest environmental impact under visible light, while undoped TiO_2_ was associated with lower impacts for UV-A-based photo-degradation. For both visible and UV-A light, Mn–TiO_2_ leads to greater potential environmental impacts. Thus, we can conclude that for MO degradation, Mn- and Ni-doping are poor choices under both UV-A and visible light irradiation. As for Co-doping, it is indeed a sustainable approach for the photo-degradation of MO under visible light. However, undoped TiO_2_ is still the best choice under UV-A irradiation. 

Our results indicate that the identity of the photocatalyst associated with greater environmental impacts changes with the employed functional unit. In this sense, Ni–TiO_2_ has a greater potential impact on human health and ecosystems, while undoped TiO_2_ has a greater impact in terms of resources when a weight-based functional unit is considered. When the LCA study considered the photocatalytic activity of the nanomaterial, Mn–TiO_2_ was found to be the catalyst with worst environmental performance among all the categories and under both visible and UV-A light. 

**Figure 4:**
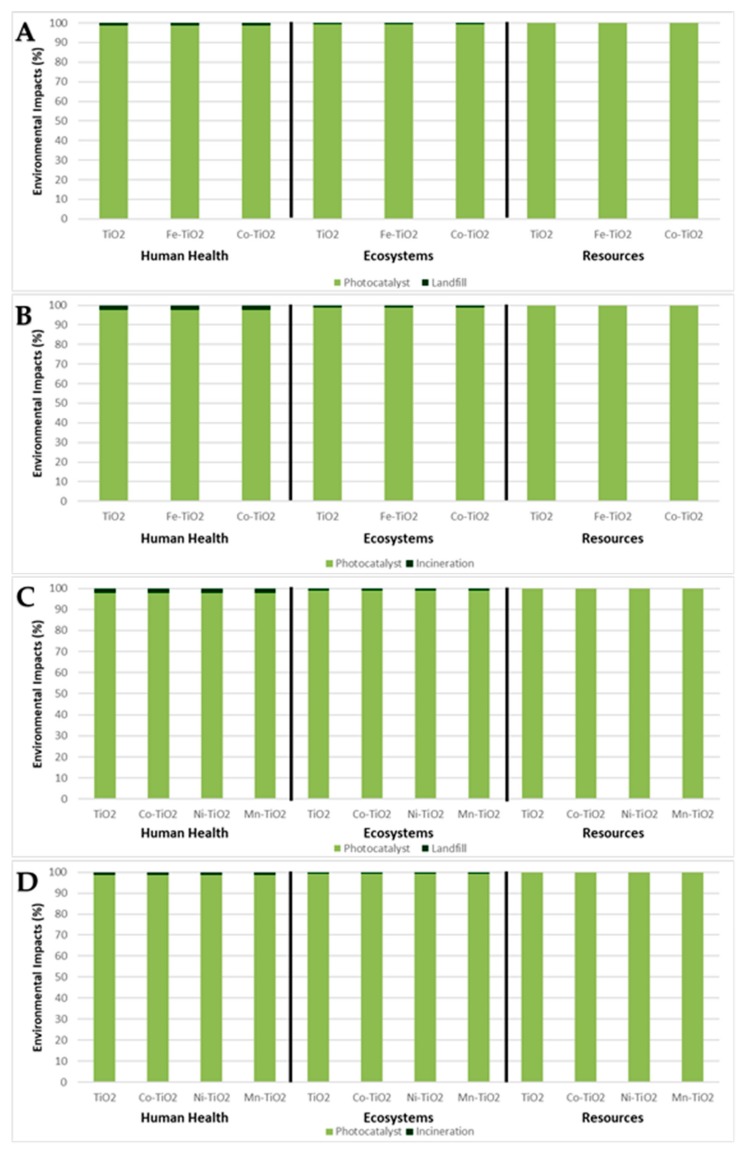
Relative environmental impacts of different TiO_2_ photocatalysts synthesis for CBZ degradation considering disposal in Landfill (**A**) or Incineration (**B**), as well for MO degradation according to Landfill (**C**) and Incineration (**D**) disposal.

In order to better understand the life cycle, two possibilities for the disposal scenario were evaluated: Landfill and Incineration (Figure 4). For both CBZ (Figure 4A,B) and MO (Figure 4C,D), incineration has a slightly greater impact than landfill disposal. However, the contribution of both landfill and incineration is quite small when compared with the synthesis stage. Therefore, it is possible to conclude that the catalyst synthesis stage represents the key part of the life cycle in terms of potential environmental impact.

### 3.3. Sensitivity Analysis

A sensitivity analysis was performed for both CBZ and MO degradation. This analysis varied both the amount (± 30%) of electricity and the raw material associated with most impacts (ISO). This was done for the photocatalysts with lower potential environmental impacts in the degradation of the organic contaminants. The substitution of the material with the greatest impact for another that can serve in the synthesis of these catalysts was also evaluated.

**Figure 5:**
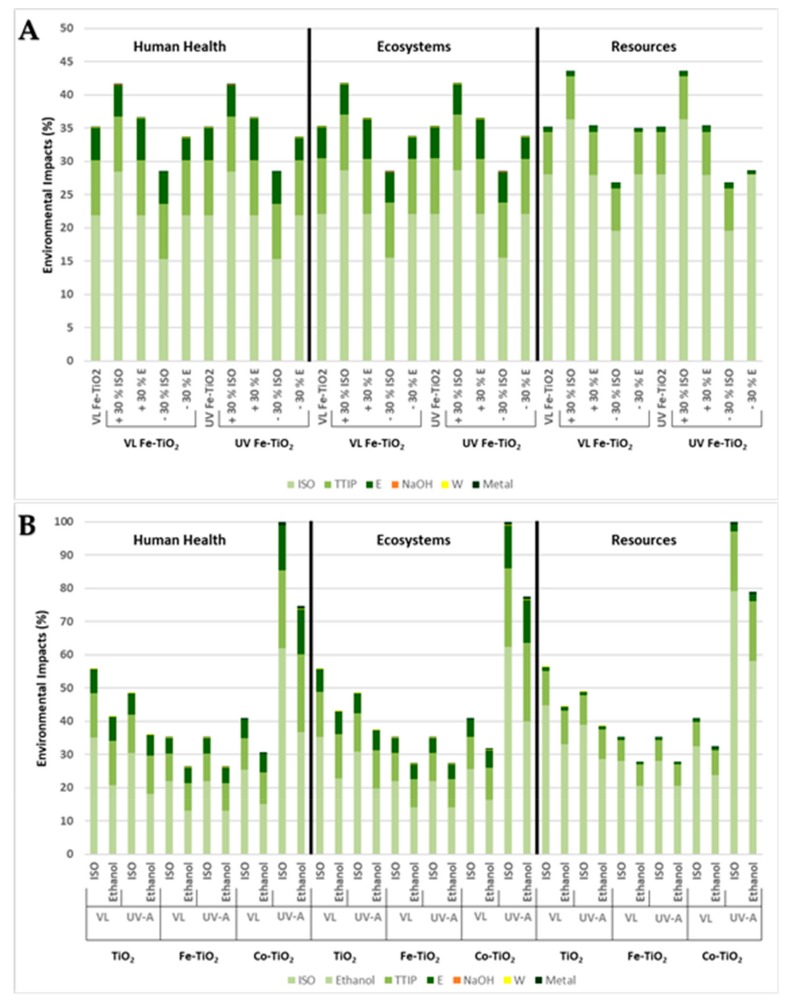
Relative environmental impacts for sensitivity analysis of Fe–TiO_2_ photocatalyst (**A**) and comparison of the substitution of ISO with Ethanol (**B**) in CBZ degradation, under either UV-A or visible-light (VL) irradiation.

For CBZ degradation, the Fe–TiO_2_ photocatalyst has the lowest environmental impact in visible and UV-A light, which motivated the sensitivity analysis regarding this catalyst (Figure 5A). In this sense, changing the amount of ISO has a significant effect on the human health (±7%), ecosystems (±7%), and resources (±8%) categories. On the other hand, varying the electricity (E) has a negligible effect on human health (i.e., an increase of 1% and a decrease of 2%), ecosystems (±1%), and an even lower effect on resources (±0.2%) impacts.

As observed, ISO is the reagent with the greatest potential environmental impact. As such, a sensitivity analysis was also performed by substituting this compound for another that has been considered in the literature for the synthesis of TiO_2_ photocatalysts, namely, ethanol [34]. The results of replacing ISO with ethanol are shown in Figure 5B. Our data indicated that, among all the photocatalysts, the use of ethanol implies less potential environmental impact in all the categories. Beyond this, a higher decrease was observed for Co–TiO_2_ under UV-A light, which presented differences higher than 20%. In fact, it has already been observed that changing the organic solvent can modify the resulting environmental impact; Wu et al. [33] found that using 1-butanol as an organic solvent has a greater environmental impact than using ISO.

**Figure 6:**
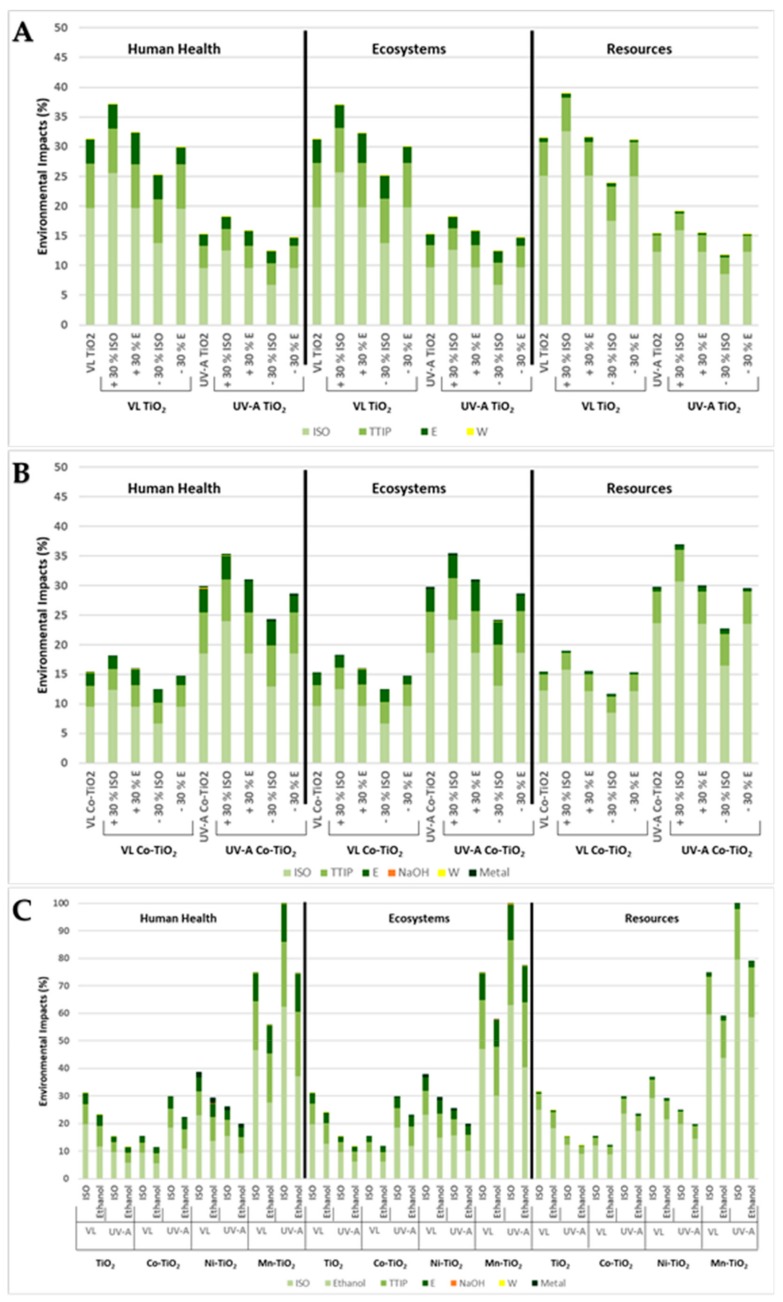
Relative environmental impact for sensitivity analysis of undoped (**A**), Co–TiO_2_ (**B**) photocatalyst and comparison of the substitution of ISO with Ethanol (**C**) in MO degradation, under either UV-A or visible-light (VL) irradiation.

For MO degradation, the photocatalyst with best performance was TiO_2_ doped with Co under visible light, and the undoped catalyst under UV-A light (Figure 6).

The results for the Co–TiO_2_ catalyst are presented in Figure 6A. When we changed the amount of ISO, we observed (under visible light) moderate effects on the human health and ecosystems (± 3%) categories, and a slightly higher effect on resources (± 4%). The same changes, but under UV-A light, had substantial influences on the human health, ecosystems (for both categories differences of ± 6%), and resources (± 8%) categories. The effects of varying the electricity (under both visible and UV-A light) were insignificant for human health, ecosystems with ± 1%, as well as for resources, with ± 0.1% (VL) and 0.2% (UV-A).

The results of the sensitivity analysis of the undoped TiO_2_ photocatalyst are shown in Figure 6B, in which the opposite behavior of Co–TiO_2_ was observed. More specifically, changing the amount of ISO under visible light has substantial effects on the human health, ecosystems, and resources categories (± 6%, ± 6% and ± 8%, respectively). Under UV-A light, a moderate influence on the human health (± 3%), ecosystems (± 3%), and resources (± 4%) categories was observed. Changing the amount of electricity still leads to insignificant effects for all categories.

The effect of replacing ISO with ethanol (Figure 6C) was also evaluated. This led to lower potential environmental impacts in all the categories, which were higher for Mn–TiO_2_ with differences greater than 15% under visible light and above 20% under UV-A light. Replacing ISO with ethanol does not change the photocatalysts with better performance relative to their environmental impact (visible light: Co–TiO_2_; UV-A: undoped TiO_2_). 

## 4. Conclusions

This study applies a first Life Cycle Assessment (LCA) to TiO_2_ photocatalysts synthesis with the purpose of evaluating the environmental performance of transition-metal-monodoped catalysts in the degradation of specific organic contaminants, Carbamazepine and Methyl Orange (respectively, a pharmaceutical and water contaminant).

Our LCA study revealed that transition-metal-monodoping has a limited environmental impact on the synthesis of TiO_2_ photocatalysts when considering a weight-based functional unit (1 kg). However, when the photocatalytic activity of each nanomaterial was taken into account, major differences were found. Thus, our results demonstrate that the comparative sustainability of monodoping with different transition metals is solely determined by their ability to enhance (or not) the photocatalytic activity of TiO_2_.

Our LCA approach also revealed that isopropyl alcohol is the reagent that has by far the highest environmental impact, with the exception of the Land Use and Mineral Resource Scarcity categories, in which the titanium precursor is the highest contributor. A sensitivity analysis indicated that varying the amount of isopropyl alcohol (± 30%) leads to important changes in the environmental profiles of the studied TiO_2_ photocatalysts. These results imply that isopropyl alcohol constitutes a critical point in the synthesis, and it should be focused on in studies aiming to increase the sustainability of producing TiO_2_ photocatalysts. Our results also indicated that replacing this chemical with ethanol increases the sustainability of these processes; therefore, further studies should be made to assess the implications of this change in the photocatalytic performance of this type of nanomaterial.

## Figures and Tables

**Table 1:** Functional unit based on photocatalytic activity for each pollutant under study. “-” refers to cases in which the photocatalysts were not used.

Photocatalyst	CBZ		MO	
	VL	UV-A	VL	UV-A
Fe–TiO_2_	1.00	1.00	-	-
Undoped TiO_2_	1.59	1.38	2.04	1.00
Co–TiO_2_	1.16	2.83	1.00	1.94
Ni–TiO_2_	-	-	2.41	1.63
Mn–TiO_2_	-	-	4.90	6.55

## Supplementary Materials

The following are available online at https://www.mdpi.com/1996-1944/13/7/1487/s1, Table S1. Amount of materials used in this LCA for each photocatalyst; Table S2. Inputs introduced in Ecoinvent database to achieve titanium precursor.
