# Supplementary material for: Life Cycle Assessment of the Sustainability of Enhancing the Photodegradation Activity of TiO2 with Metal-Doping
Source: Materials (Basel). 2020 Mar 25;13(7):1487. doi: 10.3390/ma13071487 (PMC7178238; doi:10.3390/ma13071487)
Supplement: Supplementary file 1 [file materials-13-01487-s001.pdf]

materials

Supplementary Materials

Life Cycle Assessment of the Sustainability of Enhancing the Photodegradation Activity of TiO_2 with Metal-Doping

Sónia Fernandes [1], Joaquim C.G. Esteves da Silva [1,2] and Luís Pinto da Silva [1,2,*]

[1] Chemistry Research Unit (CIQUP), Faculty of Sciences of University of Porto, R. Campo Alegre 697, 4169-007 Porto, Portugal
[2] LACOMEPHI, GreenUPorto, Department of Geosciences, Environmental and Territorial Planning, Faculty of Sciences of University of Porto, R. Campo Alegre 697, 4169-007 Porto, Portugal
* Correspondence: luis.silva@fc.up.pt

Materials used on synthesis procedures

All chemicals employed in this study were of analytic grade and used as received without further purifications.

So titanium (IV) isopropoxide ($Ti(OCH(CH_3)_2)_4$; purity > 99.99%), isopropyl alcohol (purity of 99.99%), cobaltous (II) chloride hexahydrate ($CoCl_2.6H_2O$; purity of 99.99%), manganese (II) chloride tetrahydrate ($MnCl_2.4H_2O$; purity > 99%) and iron (III) nitrate nonahydrate ($Fe(NO_3)_3.9H_2O$; purity > 98%) were purchased from Sigma Aldrich Chemicals (St. Louis, USA). On the other hand nickel (II) chloride hexahydrate ($NiCl_2.6H_2O$; purity > 97%) was acquired from Lobachimie and sodium hydroxide (NaOH; purity of 98%) from Fisher Scientific International Company (Hampton, USA).

Data inventory

Table S1. Amount of materials used in this LCA for each photocatalyst.

	TiO_2	1% Co-TiO_2	1% Ni-TiO_2	1% Mn-TiO_2	1% Fe-TiO_2
TTIP	3558.71 g	3523.12 g			
ISO	18,812.76 g	18,624.61 g			
$CoCl_2.6H_2O$	-	31.76 g	-	-	-
$NiCl_2.6H_2O$	-	-	31.95 g	-	-
$MnCl_2.4H_2O$	-	-	-	27.90 g	-
$Fe(NO_3)_3.9H_2O$	-	-	-	-	50.59 g
NaOH	-	10.68 g	10.76 g	11.28 g	15 g
H_2O	22,537.75 g	26,329.99 g	32,341.98 g	32,330.19 g	32,272.14 g
TTIP dissolution	22,537.75 g	22,312.34 g	22,312.34 g	22,312.34 g	22,312.34 g
Metal dissolution	-	2010.13 mL	4992.19 mL	4982.14 mL	4959.80 mL
NaOH dissolution	-	2007.52 mL	5037.45 mL	5035.71 mL	5000 mL
Electricity consumption	16.47 kWh	17.22 kWh			
Heating plate	1.25 kWh	2 kWh	2 kWh	2 kWh	2 kWh
Centrifuge	0.219 kWh	0.219 kWh	0.219 kWh	0.219 kWh	0.219 kWh
Oven	15 kWh	15 kWh	15 kWh	15 kWh	15 kWh
Photocatalyst produced	1000 g	1000 g	1000 g	1000 g	1000 g

TTIP was introduced based on literature [27] because it was not included on database Ecoinvent. Thus, TTIP was named Titanium (IV) isopropoxide at plant (RER).

Table S2. Inputs introduced in Ecoinvent database to achieve titanium precursor. Based on [27].

Reference Flow Amount: 1kg			
**Inventory Item**	**Amount**	**Other**	**Inventory Flow Source**
Ammonia, liquid, at regional storehouse (RER)	0.12 kg		Ecoinvent
Electricity, medium voltage, production RER, at grid (RER)	0.82 MJ	This value considered additional energy consumption	Ecoinvent
Isopropanol, at plant (RER)	0.85 kg		Ecoinvent
Titanium tetrachloride, at plant (RER)	0.67 kg		[27]
Transport, freight, rail (RER)	0.98 t-km	This value considered 600 km of freight rail transport and	Ecoinvent
Transport, lorry 16-32t, Euro3 (RER)		0.16 t-km 100 km of freight truck transport	Ecoinvent
Ammonium chloride, at plant (GLO)	0.38 kg	Co-product	Ecoinvent
Hydrochloric acid, from the reaction of hydrogen with chlorine, at plant (RER)	0.23 kg	Co-product	Ecoinvent

© 2020 by the authors. Submitted for possible open access publication under the terms and conditions of the Creative Commons Attribution (CC BY) license (http://creativecommons.org/licenses/by/4.0/).
